# Funisitis is associated with adverse neonatal outcome in low-risk unselected deliveries at or near term

**DOI:** 10.1007/s00428-015-1899-0

**Published:** 2016-02-11

**Authors:** F. A. Jessop, C. C. Lees, S. Pathak, C. E. Hook, N. J. Sebire

**Affiliations:** Department of Paediatric Pathology, Addenbrooke’s Hospital, Cambridge, UK; Departments of Paediatric Pathology, Camelia Botnar Laboratories, Great Ormond Street Hospital and Institute of Child Health, Great Ormond Street, London, WC1N 3JH UK; Department of Fetal Medicine, Addenbrooke’s Hospital, Cambridge, UK

**Keywords:** Chorioamnionitis, Funisitis, Outcome, Placenta

## Abstract

This study aimed to determine the incidence and clinical outcomes for varying patterns of placental histological inflammation (consistent with fetal or maternal inflammatory response) in an unselected population of >1000 women with a singleton pregnancy resulting in live birth delivering at or near term. One thousand one hundred nineteen cases were studied in a blind, prospective, unselected study with placentas categorized into five histological subgroups reflecting underlying maternal or fetal inflammatory response. Clinical outcomes studied included interventional delivery, an Apgar score <7 at 1 min, neonatal acidosis (pH < 7.2) and admission to neonatal special care. One hundred eighty-eight placentas (17 %) showed histological evidence of acute inflammation: 64 with funisitis (with or without other inflammation; 6 %); 16 with extensive acute inflammation across the chorionic plate, free membranes and subchorionic fibrin (1 %); 28 with acute inflammation restricted to the chorionic plate (2 %); 12 with acute inflammation restricted to the free membranes (1 %) and 68 with acute inflammation restricted to the subchorionic fibrin (6 %). Features of extensive acute inflammation were significantly associated with increased rate of interventional delivery (assisted vaginal delivery or emergency caesarean section; *P* < 0.01). The presence of funisitis was significantly associated with interventional delivery and other adverse outcomes including an Apgar score <7 at 1 min, clinical evidence of sepsis and admission to the neonatal intensive care unit (*P* < 0.05 for all). The data represent a quantitative rather than purely qualitative analysis of the contribution of histological lesions related to inflammation on short-term adverse neonatal outcomes and interventional delivery. Funisitis and extensive inflammation are associated with adverse clinical outcomes, but the precise mechanism underlying these remains to be elucidated.

## Introduction

Chorioamnionitis and funisitis are defined histologically as acute inflammatory responses in the fetal membranes and umbilical cord respectively. There is accumulated evidence that the observed acute inflammation is attributable to ascending genital tract infection [1–4], with suggestions of alternative aetiologies in rare cases [5]. The effects of inflammation in the cord and membranes are relatively well documented for both early and late infant well-being in pre-term neonates, with multiple studies reporting adverse outcomes associated with clinical and histological chorioamnionitis related to pre-term delivery [6–13].

The clinical significance of acute histological chorioamnionitis or funisitis for a neonate born at term remains uncertain. A recent cohort study of 395 women reported no association between term histological chorioamnionitis and poor neonatal outcome (Apgar score, cord blood gas analysis or admission to NICU, although this study excluded cases with clinical features of inflammation) [14], but other studies have reported associations with adverse outcomes such as early neonatal sepsis [15]. It has also previously been reported that there is no evidence of association between relatively severe term neonatal acidosis and intra-uterine infection [16]. Clinical chorioamnionitis at term has, however, been associated with increased rates of cerebral palsy [17] and has recently been reported to be associated with an increased risk of neonatal mortality [18].

Assessment of the clinical significance of acute inflammation in placentas delivered from term pregnancies is thus an important question, not least because, as guidelines for routine clinicopathological assessment of the placenta are now widespread, the reporting pathologist is required to recognize all possible pathologic lesions, while disregarding histological patterns of no or limited diagnostic significance [19]. Resolving these issues would also be of considerable interest to practising obstetricians and neonatologists in determining which placentas to submit for histological examination and in understanding the significance of the resulting histopathological findings for clinical management.

Studies in this area are generally retrospective series of clinically submitted cases and are therefore hampered by the highly pre-selected nature of such placentas which fulfil criteria for formal pathological examination in clinical practice. As part of a large study recruiting consecutive unselected low-risk pregnancies delivering at or near term, this dataset provides a rare opportunity to examine the clinical outcomes of acute inflammatory patterns, assessed in a blinded manner, in the varying compartments of placental microanatomy in this specific clinical setting.

## Methods

The parameters and methodology of the main study have been previously reported [20]: this was an unselected prospective study including 1119 subjects, approved by the Peterborough and Fenland Research and Ethics Committee (LREC Ref No 07/Q0106/51), in which women with a singleton pregnancy booking for delivery at a single maternity hospital in the East of England and delivering at or near term (97 % (1085/1119) delivered at ≥37 weeks) were recruited by written consent over a 13-month period, 2007–2008. Macroscopic placental examination was carried out either by FAJ or SP, including histological sampling, carried out in accordance with standard previously published protocols [21, 22].

Histological examination of placental, cord and membrane sections was carried out by FAJ and NJS, entirely blinded to all clinical information, cases being identified by study number only. The subgroups of chorioamnionitis used were based on previously described histological patterns reflecting either fetal or maternal inflammatory response [23, 24], as follows. In each case the most severe pattern present was recorded:

1. Funisitis (if present, this superceded any other underlying inflammatory pattern)
2. Subchorionic fibrin neutrophilic inflammation only (SCF inflammation)
3. Neutrophilic inflammation throughout the chorionic plate, free membranes and subchorionic fibrin (CP, membranes, SCF).
4. Neutrophilic inflammation of the chorionic plate (CP only)
5. Neutrophilic inflammation of free membranes (membranes only)
6. No chorioamnionitis (no CA), normal

Birth weights were routinely recorded. Markers of adverse clinical outcome in neonates included seizures, severe acidosis (pH < 7.0 on umbilical cord arterial gas at delivery) and death, in addition to less severe complications but with the potential to identify fetuses/neonates with physiological compromise. These included interventional delivery (assisted vaginal/emergency caesarean (emLSCS)), birth weight <10th percentile for gestation, an Apgar score <7 at 1 min of age, neonatal acidosis (pH < 7.2 on umbilical cord arterial gas at delivery) and admission to the neonatal special care unit (previously reported in a subset of women recruited to this study in which cord coiling was examined in relation to placental shape) [25].

Determination of mean values and standard deviations was carried out using Microsoft Excel software. OpenEpi software [26] was used to determine *P* values from *t* tests and odds ratios with confidence intervals.

## Results

One hundred eighty-eight placentas from 1119 in total included in the study (17 %) showed some degree of acute neutrophilic inflammation at any site. The clinical outcomes recorded for this overall group of 188 cases included 82 which required interventional delivery, 11 with an Apgar score <7, 12 which required NICU admission and eight with clinical sepsis. The findings for the remaining 931 deliveries with no acute placental inflammation were 175 requiring interventional delivery, 23 with an Apgar score <7, 55 requiring NICU admission and 34 showing evidence of clinical sepsis (Table 1).

**Table 1:** Clinical outcomes in histological chorioamnionitis (all cases)

	Apgar score <7 (% of cases)	Interventional delivery (% of cases)	NICU admission (% of cases)	Clinical sepsis (% of cases)
No chorioamnionitis (*n* = 931)	23 (2.5)	175 (19)	55 (5.9)	34 (3.6)
All chorioamnionitis (*n* = 188)	11 (5.8)	82 (44)	12 (6.4)	8 (4.3)

There were no significant differences in demographic characteristics between the two groups. Mean maternal age of the group with inflammation was 30 (SD 5.3) years versus 31 (SD 5.8) years for those without inflammation (*P* = 0.125). Mean gestational age at delivery of the group with inflammation was 40 (SD 1.1) weeks and 39 (SD 1.1) weeks for those without inflammation (*P* = 0.125).

For histological analysis using the criteria for the five histological subgroups as given above, 64 (5.7 % of the total) showed funisitis; 16 showed inflammation throughout the chorionic plate, free membranes and subchorionic fibrin; 28 showed inflammation restricted to the chorionic plate; 12 showed inflammation within free membranes only and 68 showed inflammation restricted to subchorionic fibrin only. There was no statistically significant difference in mean maternal age or completed gestational weeks amongst the five subgroups (data not shown).

There were no significant associations between adverse outcomes and acute inflammation in the chorionic plate alone, the free membranes alone or the subchorionic fibrin alone. Acute inflammation extending through the free membranes, chorionic plate and subchorionic fibrin was significantly associated with increased rate of interventional delivery (*P* = 0.006, OR 4.3, CI 1.5–12.1), and presence of funisitis was significantly associated with several adverse clinical outcomes (Apgar score <7 at 1 min, clinical evidence of sepsis and admission to the neonatal intensive care unit; Table 2; *P* < 0.01 for all).

**Table 2:** Subanalysis of histological subgroups against clinical outcomes with odds ratios and *P* values

	Funisitis (*n* = 64)		Chorionic plate/free membranes/subchorionic fibrin inflammation (*n* = 16)		Chorionic plate inflammation only (*n* = 28)		Free membranes only (*n* = 12)		Subchorionic fibrin only (*n* = 68)	
	*n*	OR (95 % CI), *P* value	*n*	OR (95 % CI), *P* value	*n*	OR (95 % CI), *P* value	*n*	OR (95 % CI), *P* value	*n*	OR (95 % CI), *P* value
Interventional delivery	38	6.8 (4.01, 11.8), <0.000001	8	4.3 (1.5, 12.1), 0.006	8	1.7 (0.71, 3.9), 0.21	3	1.4 (0.31, 5.16), 0.57	25	2.52 (1.48, 4.23), 0.009
Apgar score <7	7	5.0 (1.9, 11.9), 0.002	1	2.6 (0.12, 15.7), 0.40	1	1.5 (0.07, 8.4), 0.665	1	3.6 (0.16, 22.4), 0.30	1	0.59 (0.02, 3.25), 0.687
NICU admission	9	3.4 (1.3, 7.6), 0.03	0	0 (0.00, 5.7), 0.38	0	0 (0.00, 1.8), 0.19	1	2.4 (0.11, 14.7), 0.44	2	0.48 (0.07, 1.72), 0.326
Suspected neonatal sepsis	7	3.4 (1.3, 7.7), 0.01	0	0 (0.00, 3.3), 0.55	0	0 (0.00, 14.3), 0.36	1	2.4 (0.11, 14.7), 0.44	0	0 (0.00, 1.72), 0.087

## Discussion

The findings of this study have demonstrated that in an unselected population of women delivering at or near term, the presence of some types of histological placental inflammation is associated with increased rates of adverse neonatal outcome, with the majority of this increased risk being associated with those cases demonstrating histological funisitis.

There are few pre-existing studies of unselected term cohorts to offer comparison to the data presented above. Rates of chorioamnionitis overall reported at term range from 4 to 35 % [5, 14, 15, 27–30], but definitions of chorioamnionitis across studies are variable, and most lack neonatal outcome data [5, 27, 29, 30], report on a high-risk population (for example with a neonatal death rate of >6 %) [28] or show no effect on neonatal outcome but are underpowered using small populations [14]. For those studies including both histological placental assessment and neonatal outcomes, a positive association between histological chorioamnionitis and neonatal clinical sepsis has been reported in one recent study [15].

In the present study, histological features of ascending genital tract infection, including even mild cases (i.e. acute neutrophilic inflammatory cell infiltrate in one or more of free membranes, chorionic plate, subchorionic fibrin and umbilical cord), were found in around 17 % of the cases, and the frequency of funisitis was around 5 %. Patterns of acute inflammation in the placenta are often considered in terms of correlation with a fetal or maternal inflammatory response [23, 24]. Notable in this study is the finding that the adverse outcomes identified are predominantly associated with the category of a histological fetal inflammatory response (funisitis). Fetal inflammatory response syndrome (FIRS) is characterized by activation of the fetal innate immune system, secondary to intra-uterine infection/inflammation [31]. This was originally reported to be associated with raised interleukin-6 levels in umbilical cord blood [32], but as further studies have been undertaken, elevated levels of other cytokines are well described [31, 33]. The fetal inflammatory response syndrome is overwhelmingly considered in the published literature in the context of pre-term delivery [31, 32, 34], and in that setting is associated with increased frequency of both chronic lung disease [35] and adverse long-term neurodevelopmental outcome [17].

In cases of extensive placental inflammation but without funisitis, the main association was with increased rates of interventional delivery, a clinical decision which is usually based on concern for fetal well-being on the part of the clinical team caring for the labouring woman, sufficient to expedite delivery, either assisted vaginal or operative. No further information is available from this study to analyse further the precise indications for such interventional delivery in individual cases, though the decision to deliver will normally be made on the basis of an abnormal cardiotocograph (CTG) tracing of the fetal heart. Funisitis itself was significantly associated with other major adverse outcomes studied; these (Apgar score <7, suspected clinical sepsis and admission to the neonatal unit) are entirely related to fetal well-being, with suspected fetal sepsis a specific indicator for underlying possible systemic infection.

Assessing the gestational-age-independent effects of chorioamnionitis is more challenging, and may be difficult to achieve given continuing advances in neonatal care [36]. Considering the specific question of the importance of funisitis at term, given the findings in the present study, previous data is limited. Histological inflammation of the cord has been reported in term pregnancies in a small number of studies [30, 37], with uncertain clinical significance. The findings of this study support the view that ascending genital tract infection, even at term, may lead to a fetal-derived systemic inflammatory response which is associated with increased rates of potentially significant perinatal morbidity.
